# Machine learning prediction of deep vein thrombosis before staged surgery in lower-leg and ankle fractures treated with temporary external fixation

**DOI:** 10.3389/fcvm.2026.1963261

**Published:** 2026-09-30

**Authors:** Ximing Lin, Hongpeng Gao, Weizhang Lin, Shijie Wang, Zepeng Lin, Yue Yang, Peiyi Zhuang, Chenxing Cai, Rongyuan Yang

**Affiliations:** 1 Department of Orthopaedic Surgery, Zhangzhou Affiliated Hospital of Fujian Medical University, Zhangzhou, Fujian, China; 2 The Second Clinical Medical College, Fujian Medical University, Zhangzhou, Fujian, China

**Keywords:** deep vein thrombosis, lower-leg fracture, machine learning, risk prediction, shap, temporary external fixation, venous thromboembolism

## Abstract

**Background:**

Deep vein thrombosis (DVT) detected between temporary external fixation and staged definitive surgery can complicate perioperative management of lower-leg and ankle fractures. We developed machine-learning models to estimate DVT probability during this interval.

**Methods:**

This retrospective cohort included adults admitted during 2015−2025 who underwent staged fixation and bilateral lower-extremity venous ultrasonography. A stratified 70:30 split defined development and validation sets. Development-set imputation, Boruta feature selection, and five-fold cross-validation were used to develop 12 classifiers. Algorithms were compared in the validation set. The selected Naive Bayes pipeline underwent 500 development-cohort bootstrap replications for optimism correction. Discrimination, precision-recall performance, calibration, and decision curves were evaluated. SHapley additive explanations (SHAP) and a bilingual research prototype web application supported interpretation.

**Results:**

Among 356 patients, 55 (15.4%) had DVT, including 40 with calf muscular vein thrombosis. The development and validation sets comprised 250 patients (39 events) and 106 patients (16 events). Boruta retained nine predictors. Naive Bayes had the highest validation receiver operating characteristic area under the curve (AUC), 0.807 (95% confidence interval, 0.705−0.909). Precision-recall AUC was 0.343, sensitivity was 0.875, and specificity was 0.589. Beta calibration reduced the Brier score from 0.135 to 0.116 without changing discrimination. The apparent AUC was 0.858, mean bootstrap optimism was 0.084, and optimism-corrected AUC was 0.773. Injury-to-admission time, calcium, age, platelet count, fibrinogen, and hemoglobin had the largest mean absolute SHAP values.

**Conclusion:**

The model may support risk stratification for an ultrasound-detected composite DVT endpoint in this staged-treatment population. Its proposed use is to inform surveillance timing and intensity while retaining routine bilateral ultrasonography. Prospective validation is required before clinical implementation.

## 1 Introduction

Deep vein thrombosis (DVT) is a clinically consequential component of venous thromboembolism and remains an important concern after lower-extremity trauma (1, 2). Patients with severe lower-leg or ankle fractures may require temporary external fixation (3) to restore alignment and protect compromised soft tissues before staged definitive fixation. During this interval, tissue injury, inflammation, venous stasis, restricted mobility, and repeated procedures converge within a short but clinically important risk window. A newly detected thrombus may alter the timing and organization of definitive surgery, add complexity to anticoagulation and anesthesia planning, and increase concern regarding perioperative embolism or bleeding. These events are also difficult to recognize clinically because pain, swelling, warmth, and limited limb movement can be attributed to the fracture or soft-tissue injury itself. Consequently, objective venous ultrasonography frequently identifies distal axial or calf muscular vein thrombosis in patients with few specific symptoms. Pharmacologic prophylaxis reduces venous thromboembolic events in immobilized lower-limb injury populations but does not eliminate risk (4). The practical challenge is therefore not simply whether DVT can occur, but which patients warrant closer attention before definitive surgery and how routine admission information can be combined into an early, reproducible estimate of risk.

Prior studies have described substantial variation in the frequency and anatomy of DVT after orthopedic trauma. In tibial plateau fractures, prospective and retrospective cohorts have reported preoperative or in-hospital DVT burdens that depend strongly on surveillance intensity, timing, and whether calf muscular veins are included. Prospective studies found predominantly distal preoperative thromboses (5, 6), whereas postoperative or clinically important endpoints produced different frequencies (7, 8). Isolated calf muscular vein thrombosis has also been reported after tibial plateau fracture surgery (9). Studies of closed tibial shaft fractures and ankle fractures reported preoperative incidences of 13.3% and 6.4%, respectively, using routine ultrasonography (10, 11). Calcaneal fracture data similarly suggest that age and delay to ultrasound contribute to heterogeneity in observed risk (12). More recent work has distinguished ultrasound-detected thrombosis from clinically important venous thromboembolism and has drawn attention to isolated calf muscular vein thrombosis as a common but anatomically distinct endpoint (8, 9). Across these studies, candidate predictors have included age, concomitant injuries, injury or surgical delay, anemia, coagulation markers, and nutritional or metabolic measurements. However, most cohorts combined heterogeneous treatment stages, focused on a single fracture site, or used a postoperative endpoint, limiting direct application to patients specifically managed with temporary external fixation while awaiting staged definitive surgery. Temporary fixation also identifies a clinically selected subgroup: the indication often reflects swelling, contamination, instability, or soft-tissue compromise, while the interval to definitive fixation varies with recovery of the local soft-tissue envelope and the patient's general condition. Risk estimates derived from unselected operative fractures may therefore average across patients with substantially different injury burdens and observation times.

Clinical prediction work in this field has also evolved unevenly. Conventional logistic models remain common and are attractive for interpretability, yet they may inadequately represent nonlinear effects and interactions among trauma severity, time, hematologic response, and metabolic status. Machine-learning studies in lower-extremity fractures and ankle fractures have compared several algorithms, but their populations, outcome definitions, and validation designs differ materially (13, 14). A recent systematic review of perioperative DVT prediction models in lower-limb fractures identified age, D-dimer, injury-to-admission time, and injury-to-surgery time as recurrent predictors, while also noting high risk of bias, incomplete reporting of missing data, and limited external validation (15). Furthermore, discrimination alone is insufficient for judging a clinical prediction model. Precision-recall performance is informative when events are imbalanced (16). Brier score and calibration assess probability accuracy (17, 18), whereas decision-curve analysis examines threshold-specific net benefit (19). Relatively few orthopedic DVT studies report these domains together. Model explanations and accessible research interfaces are also uncommon; when used carefully, SHapley additive explanations (SHAP) can show how measured features contribute to a prediction without implying causality (20).

Accordingly, this study aimed to develop and internally validate machine-learning models for DVT detected after temporary external fixation and before staged definitive surgery in adults with lower-leg or ankle fractures. We compared candidate algorithms using discrimination, precision-recall performance, probability error, calibration, and threshold-specific net benefit; interpreted the selected model with SHAP at global and individual levels; and translated the locked model into a bilingual research prototype web application. Reporting followed TRIPOD + AI, with methodological appraisal informed by PROBAST + AI (21, 22).

## 2 Materials and methods

### 2.1 Study design and setting

This single-center retrospective cohort study analyzed routinely collected inpatient data from the Department of Orthopedic Surgery, Zhangzhou Affiliated Hospital of Fujian Medical University. Eligible admissions occurred from 1 January 2015 through 31 December 2025. The target clinical window extended from temporary external fixation to staged definitive surgery, with DVT ascertained through bilateral lower-extremity venous ultrasonography. The report follows the principles of TRIPOD + AI for prediction-model studies (21).

### 2.2 Participants

Adults aged 18 years or older were eligible when they had a tibial plateau, tibial or fibular shaft, distal tibial/pilon, or ankle fracture; underwent temporary lower-extremity external fixation after written surgical consent; subsequently underwent definitive internal fixation as the second stage of treatment; and had a bilateral lower-extremity venous ultrasound record available before definitive surgery. Concomitant injuries at other anatomic sites did not constitute an exclusion criterion. Patients were excluded for pathological fracture, a history of venous thromboembolism, oral contraceptive use within the preceding month, pregnancy, a hematologic disorder, duplicate records arising from repeated admissions or procedures, external fixation confined to a non-target site such as the femur or pelvis, or lower-leg amputation. The final screening pathway is shown in Figure 1.

**Figure 1:**
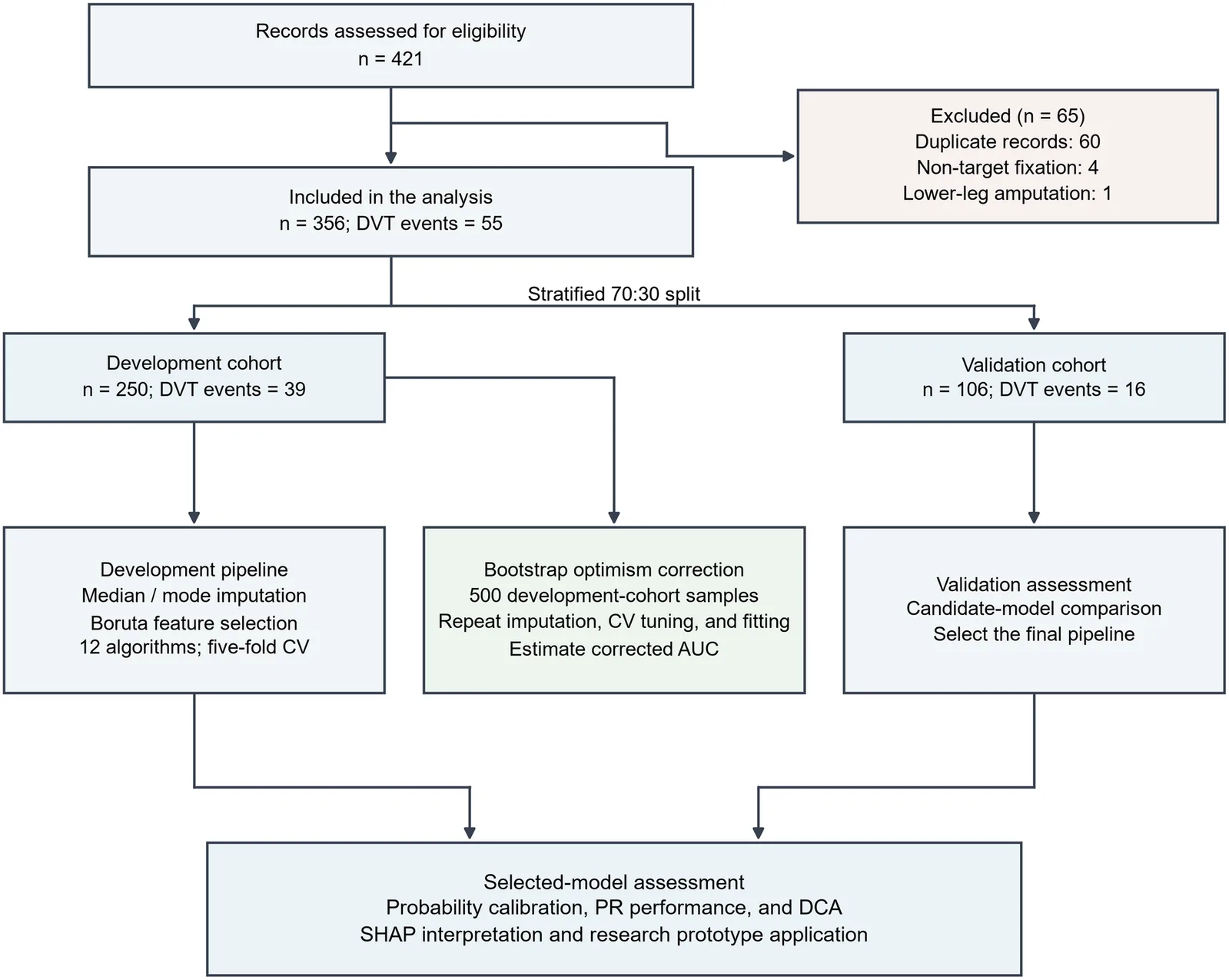
Patient selection and analysis workflow. A stratified 70:30 split defined the development and validation sets. The validation set was used for candidate-algorithm comparison and final-model selection. A separate development-cohort branch shows 500 bootstrap replications with repeated imputation, patient-grouped cross-validation for tuning, and model fitting. Bootstrap evaluation compared performance in each resampled dataset with performance in the original development cohort.

### 2.3 Perioperative management and outcome ascertainment

Temporary external fixation and definitive internal fixation were performed according to fracture pattern, soft-tissue condition, and the treating surgical team's assessment. According to the institutional clinical pathway, patients without contraindications routinely received enoxaparin 40 mg subcutaneously once daily, initiated 12 h after external fixation. Enoxaparin was continued during the interval before definitive surgery, withheld approximately 12 h before surgery, and resumed 12 h postoperatively after adequate hemostasis had been confirmed.

According to the institutional surveillance protocol, bilateral lower-extremity venous ultrasonography was routinely repeated approximately 48 h after temporary external fixation, every 5−7 days thereafter, and within 48 h before definitive internal fixation. Patients with detected DVT received treatment and reassessment of surgical timing. The outcome was any ultrasound-detected DVT during the interval between external fixation and definitive surgery, including proximal DVT, distal axial DVT, and calf muscular vein thrombosis. Isolated superficial venous thrombosis was excluded. The endpoint was defined as DVT detected during the staged-treatment interval.

### 2.4 Candidate predictors and data definitions

Variables recorded before outcome assessment included sex, admission to an intensive care or surgical monitoring unit, anesthesia type, American Society of Anesthesiologists physical status, affected side, concomitant acute injury, hypertension, diabetes mellitus, coronary heart disease, previous cerebral infarction, chronic kidney disease, chronic liver disease, malignancy, high-energy injury, open fracture, smoking history, alcohol use, age, injury-to-admission time, and body mass index. Admission laboratory variables included white blood cell, neutrophil, lymphocyte, hemoglobin, hematocrit, platelet, and mean platelet volume measurements; prothrombin time, activated partial thromboplastin time, international normalized ratio, fibrinogen, thrombin time, and D-dimer; albumin, alanine aminotransferase, aspartate aminotransferase, total bilirubin, creatinine, and blood urea nitrogen; and sodium, potassium, calcium, chloride, magnesium, and phosphorus. Neutrophil-to-lymphocyte ratio, platelet-to-lymphocyte ratio, and systemic immune-inflammation index were derived from the admission blood count. Fracture-site variables were retained for cohort description, whereas outcome anatomy, laterality, follow-up progression, and other post-outcome information were excluded from predictor development. Biochemical measurements were performed using a Beckman Coulter AU5800 analyzer; this analytical platform remained unchanged throughout the study period.

Injury energy was classified according to the documented mechanism. Road-traffic collisions, falls from height, crush injuries, machinery-related injuries, and direct impacts from heavy objects were classified as high-energy injuries. Ground-level falls, missteps on stairs, sports injuries, and other low-energy mechanisms were classified as non-high-energy injuries. Concomitant acute injury was defined as a documented acute craniocerebral, thoracic, abdominal, pelvic, spinal, or upper-extremity injury.

Admission hemodynamic status was additionally described using systolic and diastolic blood pressure, heart rate, and shock index, calculated as heart rate divided by systolic blood pressure. These measurements and the observation intervals were used for supplementary cohort description, not added to the prediction model.

### 2.5 Data partitioning, missing data, and feature selection

A stratified 70:30 split produced a development set of 250 patients (39 DVT events) and a validation set of 106 patients (16 DVT events). Prespecified transformations were applied to right-skewed continuous variables. Numeric missing values were replaced by the development-set median and categorical missing values by the development-set mode; the learned values were then applied unchanged to the validation set. Predictors with near-zero variance or deterministic redundancy were removed before feature selection. Validation outcomes contributed neither to imputation nor to feature selection.

Boruta was applied to the processed development data as the all-relevant feature-selection method (23). Variables confirmed by Boruta were carried forward as a common predictor set for model development. Feature selection was confined to the development set, and Figure 2 displays the decisions for all candidate clinical variables. LASSO logistic regression was fitted within the development data as a comparator and as one of the candidate algorithms, but it did not define the common predictor set (24).

**Figure 2:**
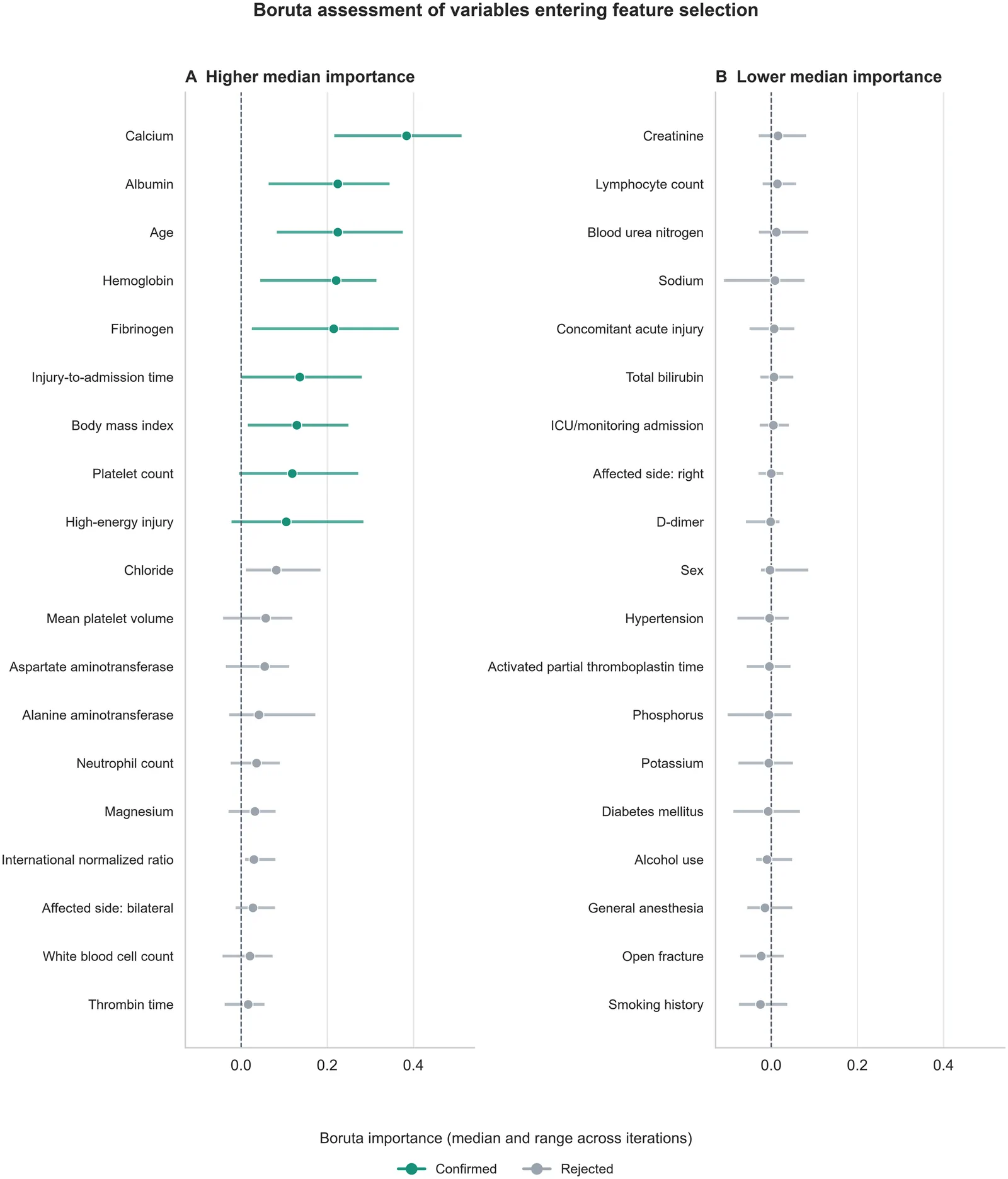
Boruta assessment of all variables entering boruta after preprocessing in the development set. Points show median importance and horizontal lines show the range across Boruta iterations. Colors indicate Confirmed and Rejected decisions determined against shadow features.

### 2.6 Model development and internal validation

Twelve candidate classifiers were fitted to the same Boruta-retained predictor set: random forest, extreme gradient boosting, radial support vector machine, logistic regression, k-nearest neighbors, partial least squares, gradient boosting machine, feed-forward neural network, Naive Bayes, linear discriminant analysis, LASSO logistic regression, and AdaBoost. Hyperparameters, where applicable, were selected using stratified five-fold cross-validation in the development set, with mean cross-validated ROC-AUC as the primary tuning criterion. PR-AUC, Brier score, sensitivity, and specificity were retained as complementary metrics. For subsequent calibration and threshold selection, development-set out-of-fold predictions were generated using stratified five-fold cross-validation repeated 10 times. Model fitting used R version 4.6.0 and the caret framework (25).

After model specifications and any applicable hyperparameters were locked, each model generated validation-set predictions. The validation set was used for candidate-algorithm comparison and final-model selection. ROC-AUC with a 95% confidence interval was calculated using the DeLong method (26). PR-AUC was reported because of the imbalanced outcome distribution (16). The Brier score quantified overall probability error; calibration intercept and slope summarized calibration-in-the-large and spread (17, 18, 27). The classification threshold was selected by maximizing the Youden index in patient-level mean probabilities from these repeated development-set out-of-fold predictions and was fixed before application to the validation set. Sensitivity and specificity were calculated using the cutoff on the original probability scale. Decision-curve analysis compared model-based strategies with treat-all and treat-none reference strategies over probability thresholds from 0.05 to 0.30 (19). This was an exploratory range for further clinical assessment, not an established clinical threshold interval or a set of treatment thresholds.

For the Naive Bayes modeling pipeline, 500 ordinary bootstrap samples of the same size as the development cohort were drawn with replacement. Within each bootstrap replicate, hyperparameters were reselected using patient-grouped stratified five-fold cross-validation, keeping all copies of each patient in the same fold. Median imputation was estimated separately within the training portion of each fold. After tuning, median imputation was re-estimated from the full bootstrap sample before final model fitting. The selected configuration was evaluated in the bootstrap sample and in the original development cohort. Apparent AUC was the development-cohort performance of the pipeline fitted to the original development data. Each optimism estimate equaled bootstrap-sample AUC minus test-on-original AUC; corrected AUC equaled apparent AUC minus mean optimism.

### 2.7 Model calibration

Calibration was evaluated after model fitting using calibration plots, the Brier score, and calibration intercept and slope. When the original probabilities showed material disagreement with observed risks, Platt scaling and monotonic three-parameter Beta calibration were fitted by maximum likelihood to development-set out-of-fold predictions generated from 10 repeats of stratified five-fold cross-validation (28). The calibrator with the lower development out-of-fold Brier score was locked and applied to validation predictions without refitting the base classifier. Validation outcomes were not used to fit or select the calibrator. Because Beta calibration is monotonic, the development-derived Youden cutoff was transformed using the locked Beta function for use with calibrated probabilities in the research prototype application.

### 2.8 Model explanation and research prototype application

SHapley additive explanations (SHAP) were used for global and individual interpretation of the selected model (29). Global importance, beeswarm, dependence, individual contribution, and force plots summarized the magnitude and direction of feature contributions and patient-level prediction profiles.

The finalized preprocessing pipeline, prediction model, and calibration procedure were implemented in a bilingual Shiny research prototype application. The prototype accepts the model variables and displays an individualized probability estimate for research-oriented preoperative risk stratification. Its intended use is to inform the timing and intensity of surveillance, not to determine whether routine bilateral venous ultrasonography is performed.

### 2.9 Statistical analysis of cohort characteristics

Descriptive tables were generated from the original, non-imputed records. Continuous variables are presented as mean ± standard deviation or median (interquartile range) according to distribution and were compared with Welch's *t*-test or the Mann–Whitney U test. Categorical variables are presented as number (percentage) and were compared with the chi-square test or Fisher's exact test, as appropriate. Two-sided *P* values below 0.05 were considered statistically significant for descriptive comparisons, which remained separate from causal inference and development-set feature selection. Observation intervals and admission vital signs were summarized as medians (interquartile ranges) and compared between outcome groups using two-sided Mann–Whitney U tests. These variables were analyzed without imputation.

## 3 Results

### 3.1 Patient selection and outcome frequency

Among 421 records assessed for eligibility, 65 were excluded: 60 duplicate observations, four records involving only femoral, pelvic, or other non-target external fixation, and one lower-leg amputation. The final cohort comprised 356 patients, of whom 55 had DVT detected before staged definitive surgery (15.4%) and 301 did not (84.6%) (Figure 1). Outcome ascertainment by bilateral lower-extremity venous ultrasonography was complete for all included patients.

### 3.2 Cohort characteristics

The cohort included 232 men and 124 women, with a mean age of 53.6 ± 15.3 years. Compared with the no-DVT group, patients with DVT were older and had lower body mass index in the unadjusted descriptive comparison (Table 1). Concomitant acute injuries were more frequent in the DVT group, whereas the recorded distribution of high-energy injury differed between groups (Table 2). Tibial/fibular shaft fractures were the most common overlapping fracture category, and tibial plateau and distal tibial/pilon fractures were more frequent among DVT-positive patients.

**Table 1:** Demographic, perioperative, and comorbidity characteristics.

Variable	Overall (*n* = 356)	No-DVT group (*n* = 301)	DVT group (*n* = 55)	*P* value
Sex				1.000
Female	124 (34.8%)	105 (34.9%)	19 (34.5%)	
Male	232 (65.2%)	196 (65.1%)	36 (65.5%)	
Age (years)	53.6 ± 15.3	52.1 ± 15.4	61.5 ± 12.2	<0.001
Body mass index (kg/m^2)	23.9 ± 3.6	24.2 ± 3.7	22.8 ± 2.9	0.004
Admission to an ICU or surgical monitoring unit	30 (8.4%)	23 (7.6%)	7 (12.7%)	0.287
General anesthesia	249 (69.9%)	207 (68.8%)	42 (76.4%)	0.332
ASA class				0.507
Class II	330 (92.7%)	277 (92.0%)	53 (96.4%)	
Class III	10 (2.8%)	9 (3.0%)	1 (1.8%)	
Class IV	16 (4.5%)	15 (5.0%)	1 (1.8%)	
Smoking history	87 (24.4%)	74 (24.6%)	13 (23.6%)	1.000
Alcohol use history	53 (14.9%)	47 (15.6%)	6 (10.9%)	0.487
Comorbidities				
Hypertension	59 (16.6%)	49 (16.3%)	10 (18.2%)	0.879
Diabetes mellitus	34 (9.6%)	30 (10.0%)	4 (7.3%)	0.707
Coronary artery disease	8 (2.2%)	7 (2.3%)	1 (1.8%)	1.000
History of cerebral infarction	11 (3.1%)	9 (3.0%)	2 (3.6%)	0.681
Chronic kidney disease	3 (0.8%)	3 (1.0%)	0 (0.0%)	1.000
Chronic liver disease	5 (1.4%)	4 (1.3%)	1 (1.8%)	0.570
History of malignancy	7 (2.0%)	6 (2.0%)	1 (1.8%)	1.000

Categorical variables are presented as n (%); continuous variables as mean ± SD or median (IQR). Descriptive statistics and tests use original non-imputed data.

**Table 2:** Injury characteristics, observation intervals, and admission hemodynamic measurements.

Variable	Overall (*n* = 356)	No-DVT group (*n* = 301)	DVT group (*n* = 55)	*P* value
Affected side				0.142
Left	172 (48.3%)	145 (48.2%)	27 (49.1%)	
Right	139 (39.0%)	122 (40.5%)	17 (30.9%)	
Bilateral	45 (12.6%)	34 (11.3%)	11 (20.0%)	
Concomitant acute injuries at other sites	191 (53.7%)	152 (50.5%)	39 (70.9%)	0.008
High-energy injury	240 (67.4%)	210 (69.8%)	30 (54.5%)	0.040
Open fracture	123 (34.6%)	106 (35.2%)	17 (30.9%)	0.643
Injury-to-admission time (hours)	3.0 (2.0, 4.0)	3.0 (2.0, 4.0)	3.0 (3.0, 4.0)	0.046
Fracture site (overlapping)				
Tibial plateau fracture	51 (14.3%)	36 (12.0%)	15 (27.3%)	0.006
Tibial/fibular shaft fracture	144 (40.4%)	121 (40.2%)	23 (41.8%)	0.940
Distal tibial or pilon fracture	79 (22.2%)	59 (19.6%)	20 (36.4%)	0.010
Ankle fracture	101 (28.4%)	89 (29.6%)	12 (21.8%)	0.313
Combined tibial and fibular fracture	307 (86.2%)	261 (86.7%)	46 (83.6%)	0.692
External fixation to definitive internal fixation (days)	11 (8, 16)	10 (7, 15)	16 (11, 23)	<0.001
External fixation to last preoperative ultrasound (days)	9.5 (6, 15)	9 (6, 13)	14 (10, 22)	<0.001
Admission systolic blood pressure (mmHg)	130 (118, 144)	130 (118, 145)	130 (118.5, 139)	0.641
Admission diastolic blood pressure (mmHg)	78 (69, 86)	78 (69, 86)	79 (67.5, 85)	0.840
Admission heart rate (beats/min)	82 (76, 93.25)	84 (76, 94)	79 (76, 88.5)	0.529
Admission shock index	0.63 (0.55, 0.73)	0.63 (0.55, 0.74)	0.62 (0.58, 0.70)	0.898

Categorical variables are presented as n (%); continuous variables as mean ± SD or median (IQR). Descriptive statistics and tests use original non-imputed data. Intervals and admission hemodynamic measurements were available for all 356 patients and were compared using two-sided Mann–Whitney U tests. Shock index was calculated as heart rate divided by systolic blood pressure. Intervals were calculated in calendar days.

Fracture-site categories overlap; one patient may occur in more than one category, so percentages may sum to more than 100%.

Admission laboratory findings are summarized in Table 3. The DVT group had lower hemoglobin, hematocrit, platelet count, albumin, calcium, and magnesium and higher prothrombin time, international normalized ratio, blood urea nitrogen, and chloride in unadjusted comparisons.

**Table 3:** Admission laboratory findings.

Variable	Overall (*n* = 356)	No-DVT group (*n* = 301)	DVT group (*n* = 55)	*P* value
Complete blood count				
White blood cell count (×10^9/L)	12.70 (10.07, 15.53)	12.96 (10.03, 15.89)	12.02 (10.22, 14.02)	0.103
Neutrophil count (×10^9/L)	10.87 (8.30, 13.63)	11.07 (8.35, 13.89)	9.90 (8.29, 12.03)	0.086
Lymphocyte count (×10^9/L)	1.11 (0.81, 1.48)	1.14 (0.82, 1.49)	0.95 (0.77, 1.31)	0.118
Hemoglobin (g/L)	115.2 ± 25.5	117.2 ± 25.6	104.3 ± 22.3	<0.001
Hematocrit (L/L)	0.343 ± 0.075	0.349 ± 0.075	0.310 ± 0.066	<0.001
Platelet count (×10^9/L)	206.0 (165.0, 254.0)	213.5 (171.0, 260.0)	172.0 (131.5, 203.5)	<0.001
Mean platelet volume (fL)	9.68 ± 1.11	9.68 ± 1.09	9.67 ± 1.20	0.979
Coagulation profile				
Prothrombin time (s)	13.05 (12.10, 13.97)	12.95 (12.00, 13.88)	13.45 (12.75, 15.18)	0.005
Activated partial thromboplastin time (s)	33.45 (29.90, 36.30)	33.15 (29.10, 36.30)	34.00 (32.70, 36.23)	0.051
International normalized ratio	1.04 (1.00, 1.14)	1.04 (0.99, 1.13)	1.09 (1.02, 1.24)	0.009
Fibrinogen (g/L)	2.73 (2.15, 3.25)	2.76 (2.21, 3.25)	2.37 (1.99, 3.09)	0.113
Thrombin time (s)	15.85 (14.80, 16.70)	15.80 (14.80, 16.68)	16.15 (14.70, 17.02)	0.506
D-dimer (ng/mL)	3,877.00 (1,384.50, 9,859.50)	3,587.00 (1,387.00, 9,341.00)	4,742.00 (1,246.50, 16,005.50)	0.237
Biochemical profile				
Albumin (g/L)	36.7 ± 7.9	37.2 ± 7.9	33.9 ± 6.9	0.002
Alanine aminotransferase (U/L)	27.8 (19.0, 41.4)	27.7 (19.0, 40.1)	28.5 (16.8, 49.5)	0.838
Aspartate aminotransferase (U/L)	33.0 (24.1, 48.3)	32.3 (24.0, 46.4)	37.2 (26.1, 63.0)	0.152
Total bilirubin (*μ*mol/L)	10.90 (7.70, 15.70)	10.70 (7.70, 15.50)	12.60 (8.45, 17.80)	0.209
Creatinine (μmol/L)	68.8 (56.1, 80.7)	68.7 (55.4, 80.4)	70.5 (58.6, 85.7)	0.570
Blood urea nitrogen (mmol/L)	5.70 (4.50, 6.70)	5.60 (4.50, 6.60)	6.45 (4.80, 7.47)	0.013
Electrolytes				
Sodium (mmol/L)	138.77 ± 3.09	138.66 ± 2.85	139.34 ± 4.15	0.259
Potassium (mmol/L)	3.89 ± 0.44	3.89 ± 0.43	3.90 ± 0.48	0.887
Calcium (mmol/L)	2.12 ± 0.18	2.14 ± 0.18	2.02 ± 0.17	<0.001
Chloride (mmol/L)	105.16 ± 3.38	104.93 ± 3.39	106.40 ± 3.10	0.003
Magnesium (mmol/L)	0.79 ± 0.12	0.80 ± 0.12	0.76 ± 0.11	0.036
Phosphorus (mmol/L)	1.02 (0.88, 1.21)	1.02 (0.88, 1.19)	1.05 (0.84, 1.25)	0.633
Derived inflammatory indices				
Neutrophil-to-lymphocyte ratio	9.65 (6.82, 14.12)	9.57 (6.63, 14.46)	9.72 (7.34, 13.26)	0.888
Platelet-to-lymphocyte ratio	185.7 (132.1, 245.7)	190.2 (133.2, 252.8)	172.1 (113.0, 197.7)	0.033
Systemic immune-inflammation index	1,976.2 (1,257.1, 3,056.0)	2,097.2 (1,283.0, 3,157.6)	1,539.4 (1,069.7, 2,520.8)	0.012

Values are based on the first laboratory measurements obtained on admission and use original non-imputed data. Values are mean ± SD or median (IQR).

The interval from external fixation to definitive internal fixation was 11 days overall (IQR 8−16; range 4–45). It was longer in the DVT group than in the no-DVT group: 16 (IQR 11–23) versus 10 (IQR 7–15) days (*P* < 0.001). The corresponding intervals to the last preoperative ultrasound were 14 (IQR 10–22) and 9 (IQR 6–13) days (*P* < 0.001). The last ultrasound was recorded 1 calendar day before surgery in 258 patients and 2 calendar days before surgery in 98 patients (Table 2).

Admission blood pressure and heart rate were available for all 356 patients. Median shock index was 0.62 (IQR 0.58–0.70) in the DVT group and 0.63 (IQR 0.55–0.74) in the no-DVT group (*P* = 0.898). Group-specific blood pressure and heart-rate summaries are presented in Table 2.

### 3.3 DVT anatomy

At first positive ultrasonography, thrombosis was left-sided in 19 patients, right-sided in 21, and bilateral in 15. Calf muscular vein thrombosis was the predominant anatomical category, accounting for 40 of 55 positive cases (72.7%); nine patients had distal axial DVT and the remainder had proximal or mixed patterns (Table 4). Calf muscular vein involvement was present in 43 patients at the first positive ultrasound and in 44 at any ultrasound during observation. Four patients showed anatomic progression on follow-up ultrasonography.

**Table 4:** DVT outcome ascertainment and anatomic distribution.

Measure	n	Percentage
Outcome ascertainment		
DVT positive	55	15.4%
DVT negative	301	84.6%
Thrombus laterality at first positive ultrasound		
Left	19	34.5%
Right	21	38.2%
Bilateral	15	27.3%
Anatomic topology at first positive ultrasound		
Calf muscular vein thrombosis	40	72.7%
Distal axial DVT	9	16.4%
Proximal DVT	1	1.8%
Mixed distal axial and calf muscular vein DVT	2	3.6%
Mixed proximal and distal axial DVT	1	1.8%
Mixed proximal DVT and superficial venous thrombosis	1	1.8%
Mixed proximal, distal axial, and calf muscular vein DVT	1	1.8%
Other thrombus anatomy measures		
Calf muscular vein involvement at first positive ultrasound	43	78.2%
Calf muscular vein involvement on any ultrasound during observation	44	80.0%
Concomitant superficial venous thrombosis	1	1.8%
Anatomic progression on follow-up ultrasound	4	7.3%

The cohort denominator is 356. Laterality and anatomy use the 55 DVT-positive patients as denominator.

Calf muscular vein thrombosis is included in the primary DVT outcome; isolated superficial venous thrombosis is not counted as DVT.

All included patients had bilateral lower-extremity venous ultrasound records available for outcome ascertainment.

### 3.4 Feature selection and model performance

Boruta confirmed nine predictors: high-energy injury, age, injury-to-admission time, body mass index, hemoglobin, platelet count, fibrinogen, albumin, and calcium (Figure 2). The same predictor set was provided to all 12 algorithms.

Validation ROC-AUC values for the six highest-ranked algorithms were 0.807 for Naive Bayes, 0.775 for LASSO logistic regression, 0.744 for gradient boosting machine, 0.727 for random forest, 0.727 for partial least squares, and 0.727 for linear discriminant analysis (Figure 3). The Naive Bayes 95% confidence interval was 0.705–0.909. Its validation PR-AUC was 0.343 and its uncalibrated Brier score was 0.135. The uncalibrated cutoff selected by the development-set Youden index was 0.0941; applying the locked Beta transformation yielded the corresponding calibrated cutoff of 0.1373 (13.7%). Because this transformation is monotonic, the two cutoffs define the same classification; validation sensitivity was 0.875 and specificity was 0.589. Naive Bayes was carried forward for calibration, interpretation, and application development. Hyperparameters for all models are provided in Supplementary Table S1.

**Figure 3:**
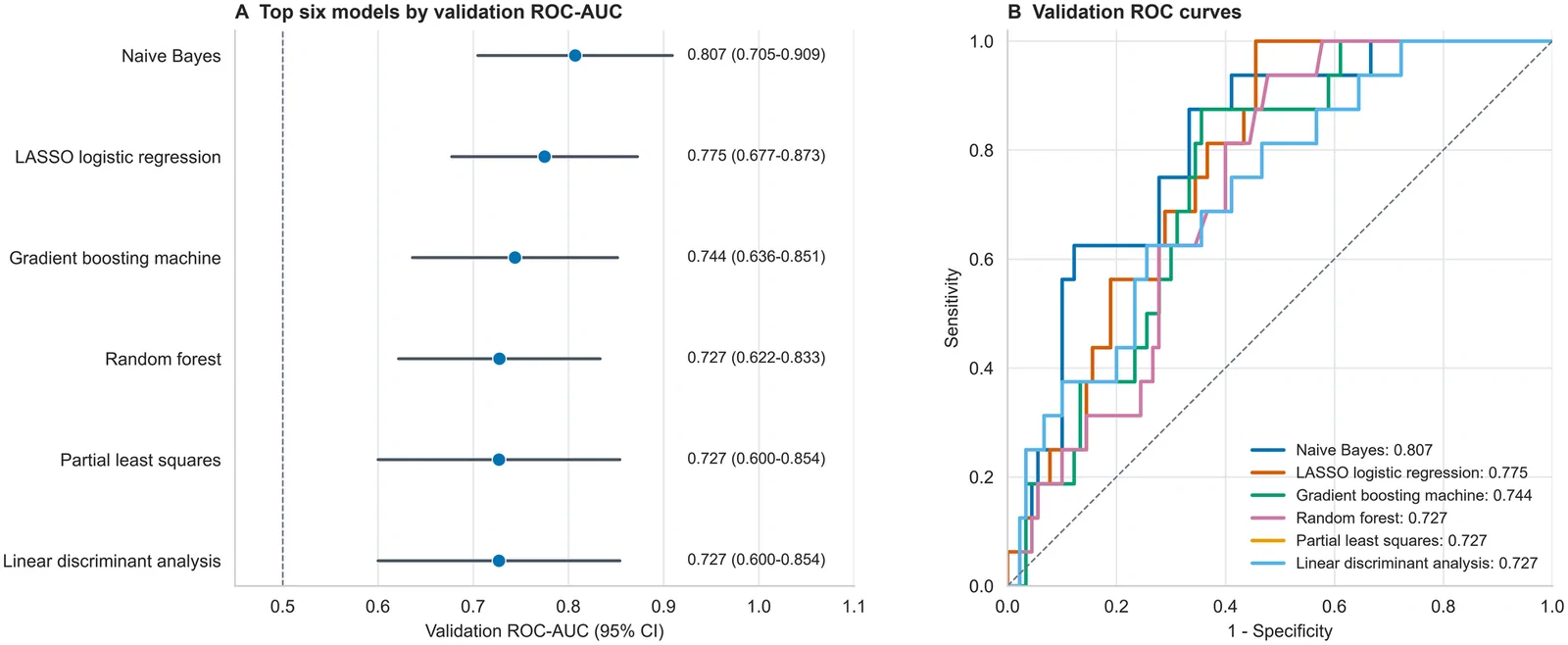
Discrimination of the six highest-ranked candidate models in the validation set. **(A)** Validation ROC-AUC with 95% confidence intervals. **(B)** Receiver operating characteristic curves with numeric AUC values. AUC, area under the receiver operating characteristic curve.

Across 500 bootstrap replications, the apparent AUC was 0.858 and mean optimism was 0.084, yielding an optimism-corrected AUC of 0.773. The bootstrap summary is provided in Supplementary Table S2.

### 3.5 Calibration and decision-curve analysis

The original Naive Bayes predictions showed a validation calibration intercept of -0.833 and slope of 0.495. Beta calibration preserved the ROC-AUC and PR-AUC while reducing the validation Brier score from 0.135 to 0.116; the resulting calibration intercept was -0.126 and slope was 1.425. Calibration plots and decision curves for original and Beta-calibrated predictions are presented in Figure 4. The model showed net benefit relative to the treat-all and treat-none reference strategies over portions of the exploratory 0.05–0.30 threshold range.

**Figure 4:**
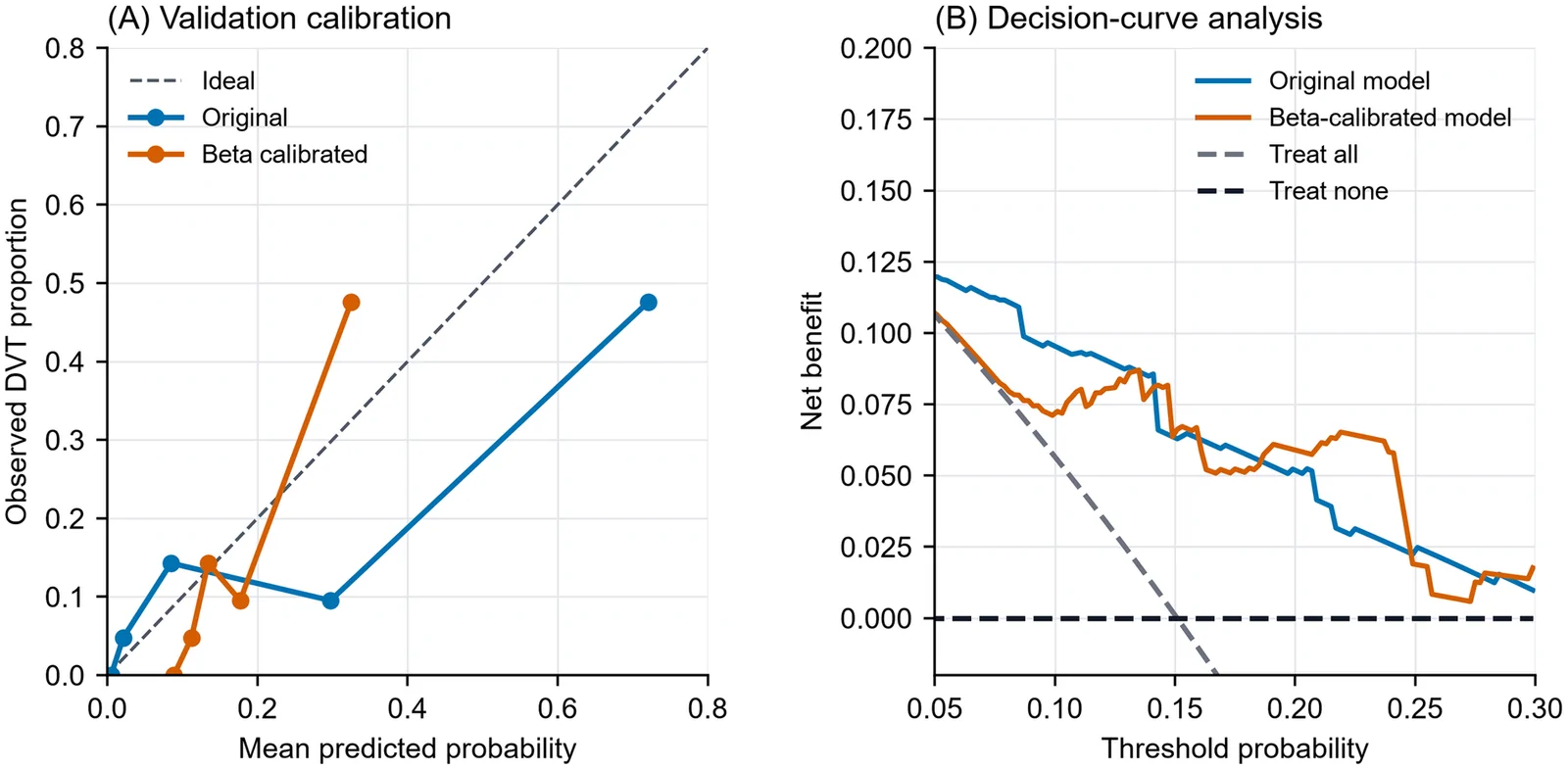
Validation calibration and decision-curve analysis for the naive Bayes model. **(A)** Original and Beta-calibrated probabilities; the 45-degree line denotes ideal calibration. **(B)** Net benefit for original and Beta-calibrated predictions compared with treat-all and treat-none strategies. The displayed threshold range of 0.05−0.30 is exploratory, not an established clinical threshold interval. Probability calibration did not alter ROC-AUC.

### 3.6 SHAP explanation

On the original Naive Bayes probability scale, the largest mean absolute SHAP values were observed for injury-to-admission time (0.070), calcium (0.063), age (0.050), platelet count (0.046), fibrinogen (0.040), and hemoglobin (0.031), followed by body mass index, albumin, and high-energy injury (Figure 5). The beeswarm and dependence plots showed variation in contribution direction and magnitude across validation observations (Figures 5, 6). In Figure 7, each individual contribution plot displays the magnitude and direction of feature-specific SHAP values for one validation observation, while the corresponding force plot presents the same upward and downward contributions along a single probability axis.

**Figure 5:**
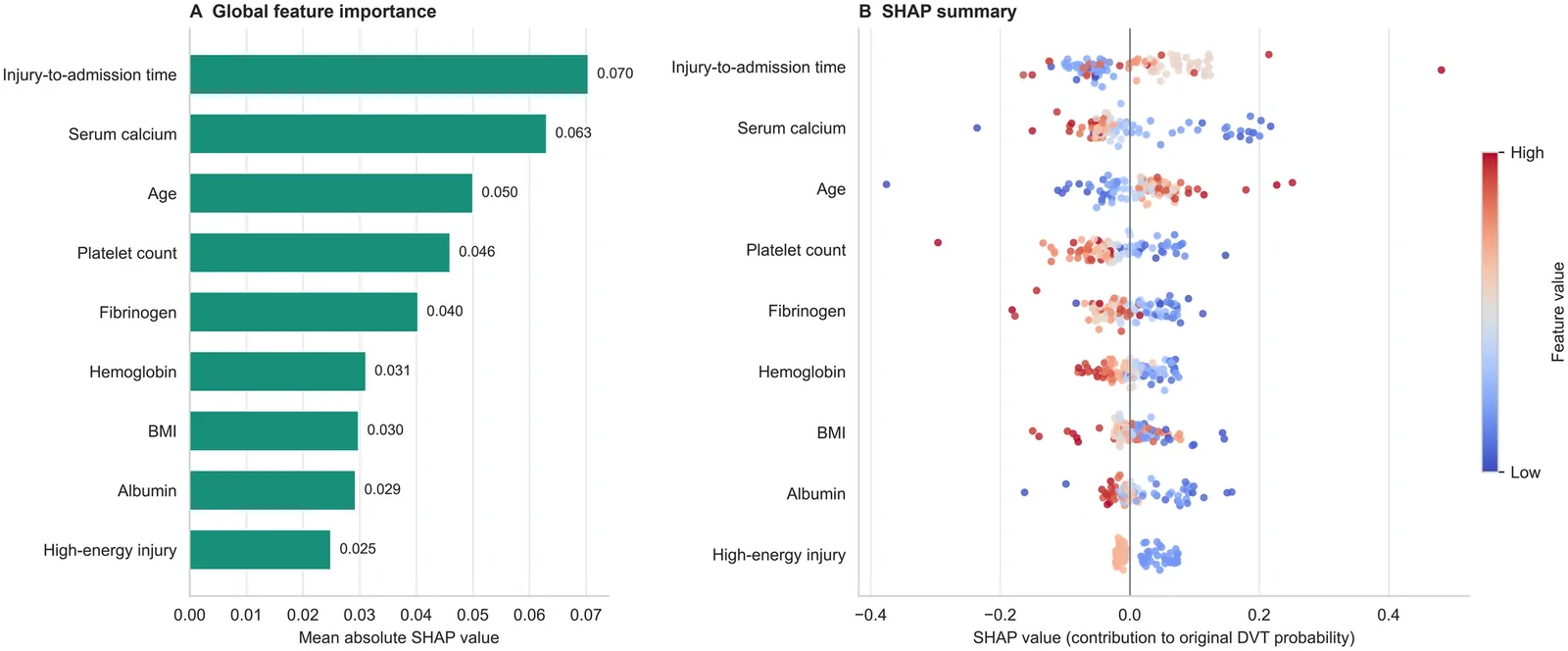
Global SHAP explanation of the locked naive Bayes model in the validation set. **(A)** Mean absolute SHAP values rank contribution magnitude. **(B)** Beeswarm plot shows the distribution and direction of feature contributions. SHAP values refer to the original uncalibrated model-predicted probability and do not represent causal effects.

**Figure 6:**
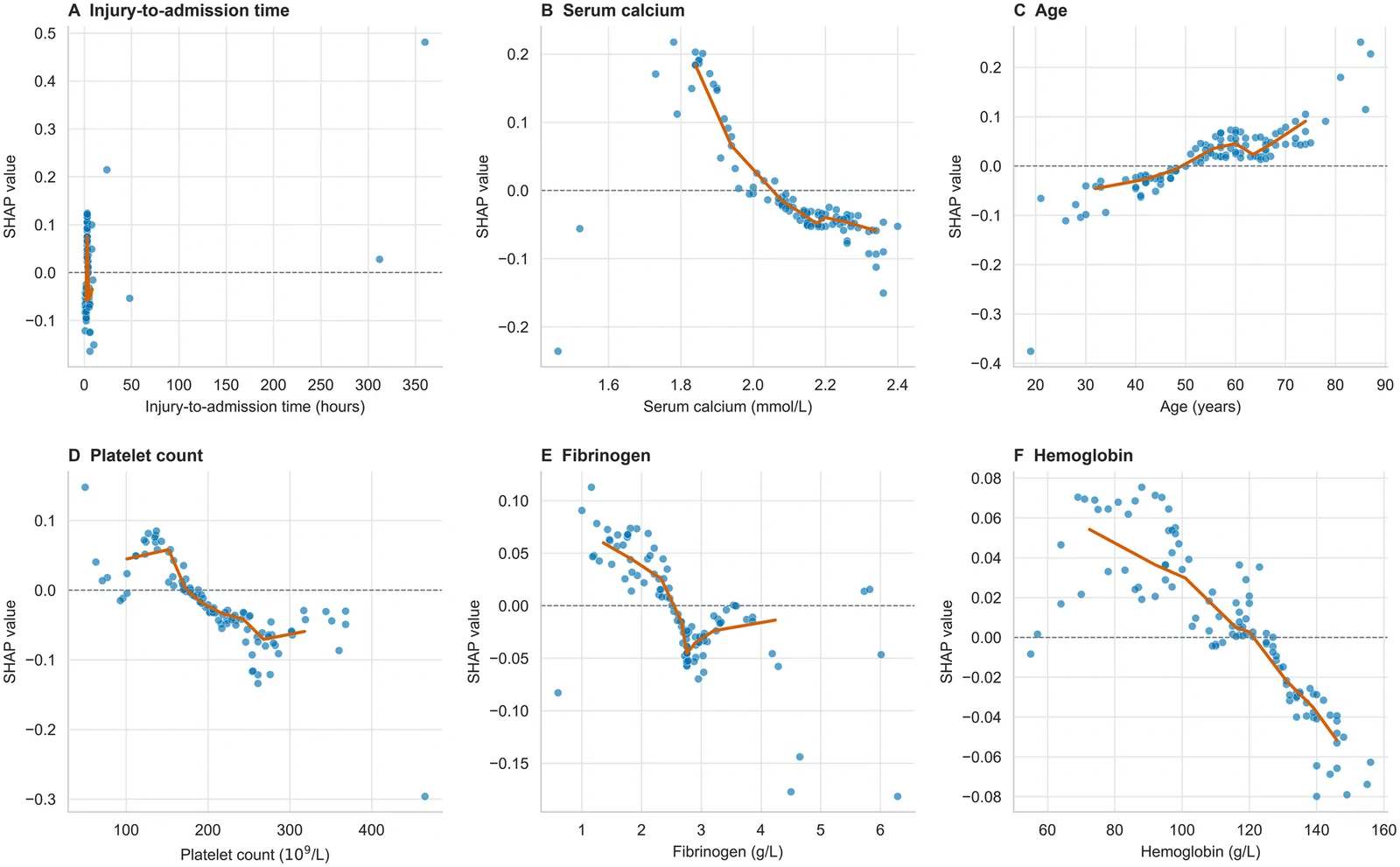
SHAP dependence plots for the six highest-ranked features. Each point represents a validation observation. The vertical axis is the SHAP contribution to the original Naive Bayes probability; the patterns describe model-specific associations rather than adjusted or causal effects.

**Figure 7:**
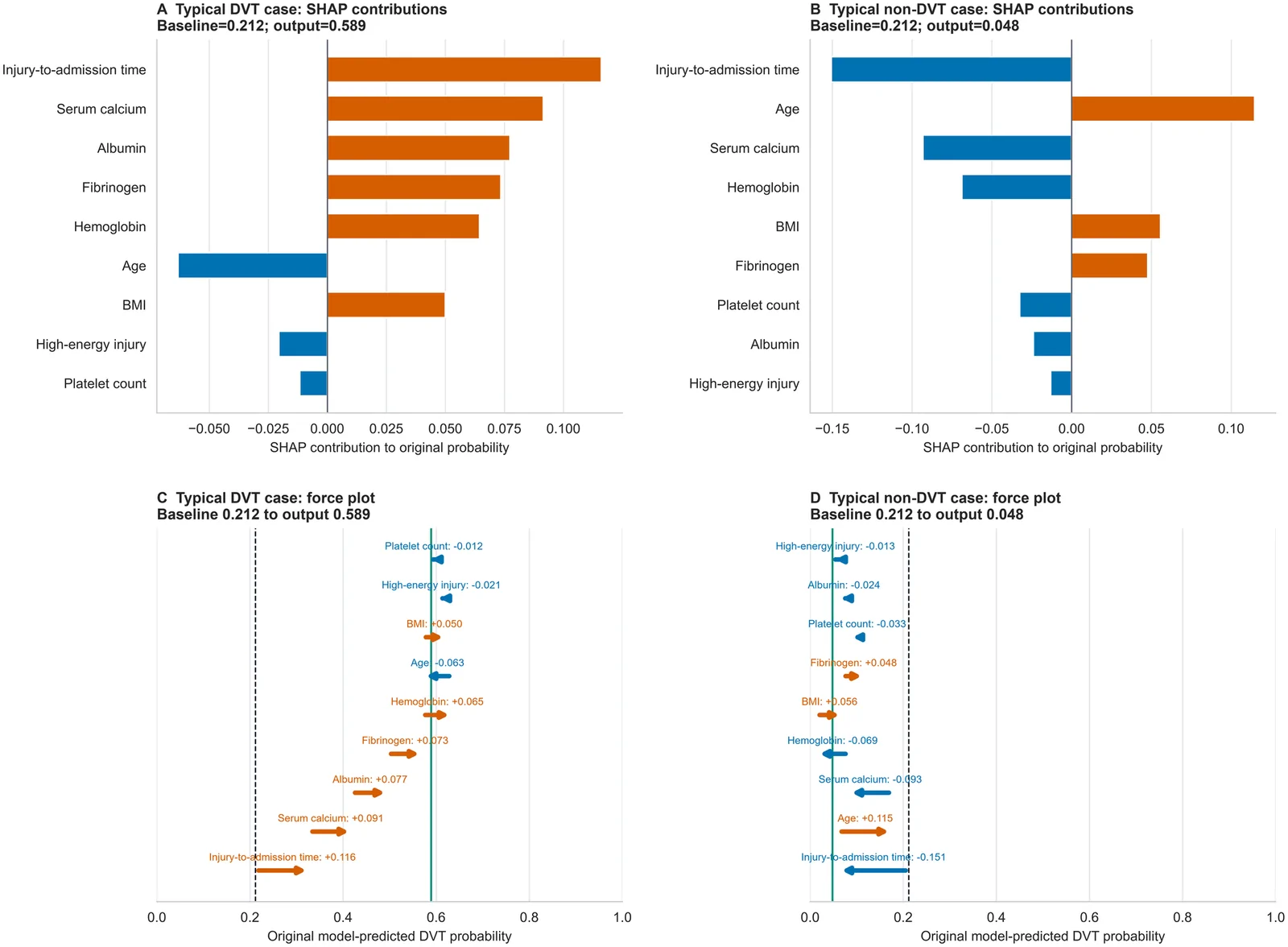
Individual SHAP explanations for representative DVT and non-DVT validation observations. Panels A and B show feature-specific SHAP contribution plots; positive and negative values indicate contributions toward higher and lower predicted probabilities, respectively. Panels C and D present the same individual explanations as force plots on the original model-predicted probability axis. No patient identifiers are displayed.

### 3.7 Research prototype application

The bilingual research prototype application implements the locked preprocessing pipeline, Naive Bayes model, and Beta calibrator. Figure 8 illustrates a hypothetical entry and the resulting Beta-calibrated model-predicted probability. The interface also reports the uncalibrated probability and provides an adjunctive estimate that can support preoperative risk stratification and consideration of earlier clinical reassessment.

**Figure 8:**
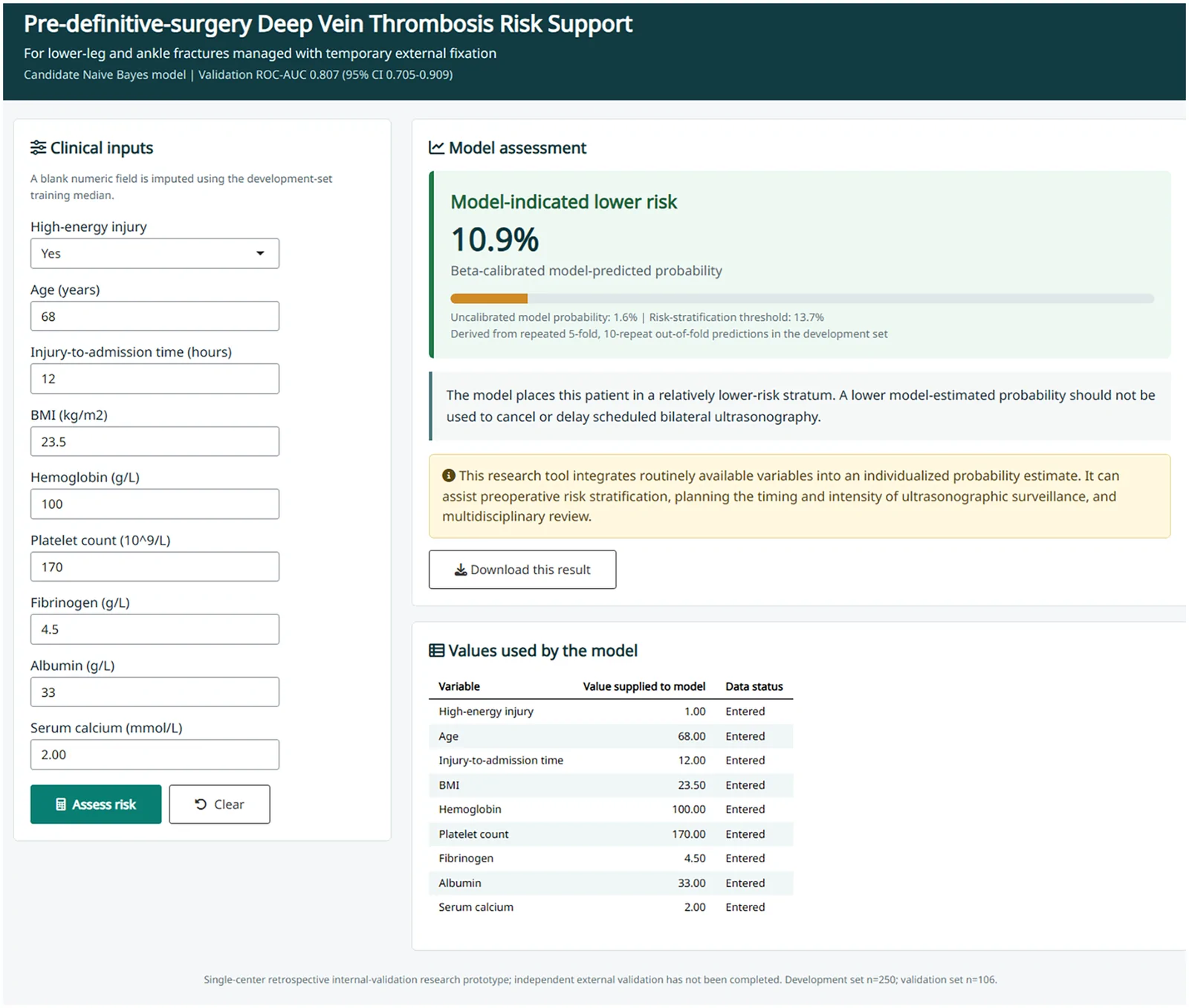
English-language interface of the research prototype application showing a hypothetical input and the resulting model output. The prototype reports a Beta-calibrated model-predicted probability together with the uncalibrated probability and provides adjunctive support for preoperative risk stratification and consideration of earlier clinical reassessment. The displayed 13.7% risk-stratification threshold is the Beta-calibrated equivalent of the uncalibrated development-set Youden cutoff (0.0941).

## 4 Discussion

This study examined DVT detected during the interval between temporary external fixation and definitive surgery in patients with lower-leg or ankle fractures. DVT was identified in 15.4% of patients. Among 12 candidate algorithms, Naive Bayes had the highest observed validation ROC-AUC. Its nine routinely available predictors combined information on admission delay, age, injury mechanism, and laboratory findings to support individualized risk stratification.

The observed frequency lies within the range reported after lower-extremity fracture, although comparisons depend on imaging schedules and outcome definitions. Routine preoperative ultrasonography detected DVT in 13.3% of closed tibial shaft fractures and 6.4% of ankle fractures (10, 11). Tibial plateau cohorts using protocolized imaging have reported larger burdens of predominantly distal thrombosis (5, 6), whereas studies restricted to postoperative or clinically important events reported lower frequencies (7, 8). In the present cohort, routine bilateral surveillance and inclusion of calf muscular vein thrombosis broadened ascertainment across the presurgical interval.

Calf muscular vein thrombosis accounted for 40 of the 55 positive cases. These thrombi involve the gastrocnemius or soleal venous systems and were also common in a study of tibial plateau fracture surgery (9). Their clinical implications differ from those of proximal or distal axial DVT. Accordingly, the model should be interpreted as predicting the composite ultrasound-detected endpoint. Only 15 events fell outside the calf-muscular-vein category, limiting the precision of anatomy-specific performance estimates. No formal subgroup performance analysis was undertaken, and performance for proximal or distal axial DVT specifically has not been established.

Temporary external fixation restores skeletal alignment and stability while the soft-tissue envelope recovers, but weight bearing and ankle motion often remain restricted during staged treatment. The resulting reduction in calf-muscle pumping can sustain venous stasis despite thromboprophylaxis (3, 4). Patients with DVT had longer intervals from external fixation to definitive surgery. A longer interval may increase exposure to limited mobility, while DVT detection and anticoagulant treatment may in turn influence surgical timing. This bidirectional relationship supports continued surveillance when definitive fixation is delayed.

Injury-to-admission time had the largest mean absolute SHAP value. Most patients were admitted within a relatively short interval, but longer delays generally shifted predictions toward a higher DVT probability, with larger contributions at prolonged intervals. Injury-to-admission time has also emerged as a recurrent predictor in lower-limb fracture models (15). A recent hip-fracture cohort reported a nonlinear association between this interval and DVT already present at admission (30). Clinically, delay may extend exposure to pain-limited mobility, ineffective calf-muscle pumping, tissue swelling, and venous stasis before structured prophylaxis and surveillance begin. It may also capture transfer complexity and delayed access to specialist care. Accurate recording of the injury time and early identification of patients with prolonged prehospital or interhospital intervals could improve the timing of venous assessment.

Serum calcium followed injury-to-admission time in SHAP importance. Lower total calcium values generally contributed to higher predicted probabilities. Calcium participates in several coagulation-complex reactions and supports platelet activation and fibrin formation. After trauma, calcium status can be influenced by hemorrhage, fluid resuscitation, citrate exposure, hypoalbuminemia, and the acute physiologic response. A multicenter trauma registry identified a nonlinear relationship between ionized calcium disturbances and coagulopathy, transfusion requirements, and mortality (31). The present model used total rather than ionized calcium, so the observed contribution probably integrates coagulation, nutritional, and resuscitation-related information. Its value is primarily practical: total calcium is routinely measured and may help recognize a physiologically vulnerable subgroup when interpreted together with albumin and the other model variables.

Age showed a broadly increasing contribution to predicted DVT probability. Prospective studies of tibial shaft and calcaneal fractures have likewise associated older age with preoperative DVT (10, 12). Several clinical mechanisms may converge in older patients, including reduced baseline mobility, less effective calf-muscle pumping, endothelial dysfunction, a greater comorbidity burden, and slower recovery after soft-tissue injury. Older patients may also remain immobile for longer while swelling subsides before definitive fixation. Age therefore provides more than demographic description in this setting. It summarizes several physiologic and functional domains that are not captured by a single laboratory measurement and is immediately available at the first assessment.

Platelet count was the fourth variable in the SHAP ordering. Lower counts, particularly within the low-to-middle observed range, tended to contribute toward higher model-predicted risk, whereas the relationship flattened at higher values. Platelet count after trauma should not be interpreted as a simple linear marker of thrombosis. Consumption at sites of tissue injury, bleeding, hemodilution, and inflammatory activation can lower the circulating count while the remaining platelets participate in hemostasis and thromboinflammatory signaling. A lower-limb fracture study also retained platelet count in a preoperative DVT prediction model (32). Here, platelet count may summarize the balance between injury-related consumption and subsequent prothrombotic activation rather than indicate that thrombocytopenia itself causes venous thrombosis.

Fibrinogen displayed a nonlinear contribution pattern. Lower and low-normal values were associated with higher predicted probabilities in much of the observed range, whereas values around the central range contributed less; the sparse extremes were more variable. Fibrinogen is both the substrate for fibrin formation and an acute-phase protein. Early after trauma, consumption and dilution may reduce its concentration, while inflammation can subsequently increase hepatic production. This time-dependent response helps explain why a single admission value need not follow a simple higher-is-worse gradient. Previous lower-limb fracture research found fibrinogen informative for preoperative DVT prediction (32). Its role in the current model is best understood as a marker of the evolving balance between coagulation, consumption, and inflammation.

Hemoglobin completed the six most influential variables. Lower concentrations generally contributed to higher predicted probabilities. A large multicenter study of tibial plateau fractures also associated lower hemoglobin with in-hospital DVT (33). In trauma care, hemoglobin can reflect pre-existing anemia, acute blood loss, hemodilution, occult bleeding, and overall injury burden. Red cell concentration also influences blood rheology and the interaction of cellular elements within a forming thrombus, although an admission hemoglobin value cannot separate these mechanisms. Clinically, the variable is useful because it is available early and may identify patients with greater physiologic disturbance. Its predictive contribution should support integrated risk assessment rather than be interpreted as evidence that anemia alone produces DVT.

Body mass index contributed less than the six variables above. Prior lower-extremity fixation research associated obesity with adverse outcomes including DVT (34), whereas DVT-positive patients in this cohort had a lower mean body mass index. Retaining body mass index as a continuous variable allowed the model to capture this cohort-specific pattern without imposing a single obesity threshold.

Albumin may summarize nutritional reserve, inflammation, capillary leakage, and dilution. Hypoalbuminemia has been associated with preoperative DVT in hip-fracture populations (35). Its contribution here likely reflects general physiologic vulnerability and also provides context for interpreting total serum calcium.

High-energy injury had the smallest mean absolute SHAP value among the retained predictors. Previous studies have reported differing associations between injury energy and preoperative thrombosis in hip and tibial plateau fractures (36, 37). Within this model, injury energy adds concise information about the trauma context and complements age, admission delay, and laboratory indicators.

High-energy injury and admission hemodynamic status represent related but distinct dimensions of trauma severity. Even when blood pressure and heart rate are relatively preserved at admission, high-energy mechanisms may be accompanied by extensive tissue injury, endothelial activation, coagulation responses, and prolonged restriction of mobility. In this cohort, the admission shock index was similar between patients with and without DVT (0.62 vs. 0.63, *P* = 0.898), indicating that this single measure of initial hemodynamic status did not distinguish the two groups. High-energy injury may therefore capture aspects of trauma burden that are not fully reflected by the admission shock index. Admission hemoglobin is also influenced by blood loss, fluid resuscitation, and sampling time and should not be interpreted as a surrogate diagnosis of hemorrhagic shock. In a recent cohort of transfused trauma patients, an elevated prehospital shock index was independently associated with VTE (38), although that population differed from the staged orthopaedic cohort examined here. Taken together, injury mechanism, admission hemodynamic status, and hemoglobin reflect complementary dimensions of trauma severity and should not be interpreted interchangeably.

The validation ROC-AUC of 0.807 indicates useful discrimination in the validation cohort. The bootstrap-corrected AUC of 0.773 provides an additional estimate of discrimination after adjustment for optimism in the fitted Naive Bayes pipeline. Together, these findings support the stability of the model's discriminatory performance and suggest that the observed validation performance was not solely attributable to model optimism. PR-AUC complements ROC-AUC under the observed event frequency (16), whereas the Brier score and decision-curve analysis provide complementary information on overall probabilistic accuracy and potential clinical net benefit, respectively (18, 19).

The original probabilities were imperfectly calibrated. Beta calibration reduced probability error while preserving ROC-AUC and PR-AUC (28), supporting clearer use of predicted probabilities for risk grouping and communication in the research prototype application.

SHAP made the fitted relationships inspectable at both cohort and patient levels (29), consistent with its use in venous thrombosis prediction research (20). Within the existing ultrasound pathway, a higher predicted probability could inform earlier clinical reassessment, earlier ultrasonography, and, when clinically indicated, additional repeat ultrasonography. A lower probability would not alter scheduled bilateral surveillance. Across the exploratory 0.05–0.30 threshold range, decision-curve analysis indicated potential net benefit under different true-positive and false-positive trade-offs. These findings support prospective evaluation of the model as an adjunct to routine surveillance.

This study focused on a defined interval between temporary external fixation and definitive surgery, used routine bilateral ultrasonography for outcome ascertainment, and compared 12 algorithms using a common predictor set. Model evaluation encompassed discrimination, precision-recall performance, calibration, decision-curve analysis, model explanation, and bootstrap optimism correction. The retrospective single-center design and limited number of events constrain the precision and stability of the estimates. Differences in observation duration may have affected opportunities for DVT detection, and the available dates did not permit time-adjusted validation. Because ultrasonography before external fixation was not uniformly performed, the endpoint was DVT detected during staged treatment rather than DVT specifically attributable to external fixation. Mobility and soft-tissue severity were incompletely characterized. The findings are most directly applicable to patients with lower-leg or ankle fractures managed with staged fixation. Prospective multicenter studies with standardized imaging and care-process variables are warranted to evaluate model performance and clinical implementation.

## 5 Conclusion

A machine-learning approach integrating nine routinely available variables provided individualized estimates of ultrasound-detected DVT probability during staged treatment of lower-leg and ankle fractures. Among the evaluated algorithms, the selected Naive Bayes pipeline showed useful discrimination, while Beta calibration improved probability accuracy and SHAP described individual feature contributions. The bilingual research prototype application may support risk stratification and decisions about surveillance timing and intensity within routine bilateral ultrasonography. Prospective validation is required before routine implementation.

## Data Availability

The datasets presented in this article are not readily available because the dataset is not publicly available due to institutional regulations and patient privacy considerations. De-identified data may be made available by the corresponding author upon reasonable request, subject to approval by the relevant institutional and ethics committee and in accordance with applicable data protection regulations. Requests to access the datasets should be directed to Hongpeng Gao, decadehp@163.com.
